# Composition of Brazil Nut (*Bertholletia excels* HBK), Its Beverage and By-Products: A Healthy Food and Potential Source of Ingredients

**DOI:** 10.3390/foods10123007

**Published:** 2021-12-04

**Authors:** Wilson V. Vasquez-Rojas, Diana Martín, Beatriz Miralles, Isidra Recio, Tiziana Fornari, M. Pilar Cano

**Affiliations:** 1 Department of Biotechnology and Microbiology of Foods, Institute of Food Science Research, 28049 Madrid, Spain; ing.valeriovasquez@gmail.com; 2 Department of Production and Characterization of Novel Foods, Institute of Food Science Research, 28049 Madrid, Spain; diana.martin@uam.es (D.M.); tiziana.fornari@uam.es (T.F.); 3 Department of Bioactivity and Food Analysis, Institute of Food Science Research, 28049 Madrid, Spain; beatriz.miralles@csic.es (B.M.); i.recio@csic.es (I.R.)

**Keywords:** Brazil nut, cake (by-product), Brazil nut beverage, phenolic compounds, fatty acids, proteins, tocopherols, squalene, antioxidant capacity, minerals, selenium

## Abstract

The consumption of plant-based beverages is a growing trend and, consequently, the search for alternative plant sources, the improvement of beverage quality and the use of their by-products, acquire great interest. Thus, the purpose of this work was to characterize the composition (nutrients, phytochemicals and antioxidant activity) of the Brazil nut (BN), its whole beverage (WBM), water-soluble beverage (BM-S), and its by-products of the beverage production: cake, sediment fraction (BM-D), and fat fraction (BM-F). In this study, advanced methodologies for the analysis of the components were employed to assess HPLC-ESI-QTOF (phenolic compounds), GC (fatty acids), and MALDI-TOF/TOF (proteins and peptides). The production of WBM was based on a hot water extraction process, and the production of BM-S includes an additional centrifugation step. The BN showed an interesting nutritional quality and outstanding content of unsaturated fatty acids. The investigation found the following in the composition of the BN: phenolic compounds (mainly flavan-3-ols as Catechin (and glycosides or derivatives), Epicatechin (and glycosides or derivatives), Quercetin and Myricetin-3-O-rhamnoside, hydroxybenzoic acids as Gallic acid (and derivatives), 4-hydroxybenzoic acid, ellagic acid, Vanillic acid, p-Coumaric acid and Ferulic acid, bioactive minor lipid components (β-Sitosterol, γ-Tocopherol, α-Tocopherol and squalene), and a high level of selenium. In beverages, WBM had a higher lipid content than BM-S, a factor that influenced the energy characteristics and the content of bioactive minor lipid components. The level of phenolic compounds and selenium were outstanding in both beverages. Hydrothermal processing can promote some lipolysis, with an increase in free fatty acids and monoglycerides content. In by-products, the BM-F stood out due to its bioactive minor lipid components, the BM-D showed a highlight in protein and mineral contents, and the cake retained important nutrients and phytochemicals from the BN. In general, the BN and its beverages are healthy foods, and its by-products could be used to obtain healthy ingredients with appreciable biological activities (such as antioxidant activity).

## 1. Introduction

The Brazil nut (*Bertholletia excelsa* HBK), also known as the Amazon nut, is a plant of the family *Lecythidaceae*, native to the Amazon rainforest and concentrated along the Amazon basin, marketed mainly by the countries of Bolivia, Brazil, and Peru. The socioeconomic and environmental importance of the Brazil nut lies in its production system as a non-timber forest product coming mainly from natural areas (not crops) and the generation of income in local populations [1].

In the nutritional and health field, in the last decade, there has been an increased interest in nuts and their positive effects on human health, associating nut consumption with the reduced risk of various degenerative diseases [2]. Studies on Brazil nut consumption, and its effects on health, report interesting benefits, such as the reduction in risk factors for cardiovascular disease, cancer prevention, and improved cognitive functions. These benefits can be attributed to the biological properties, nutrients, and phytochemical constituents that this nut possesses, such as its majority level of unsaturated fatty acids, selenoamino acids, dietary fiber, minerals, phenolic compounds, tocopherols, and phytosterols [3].

Considering these potential benefits of the Brazil nut, its added value has been encouraged with a focus on the development of health-promoting foods, such a Brazil nut beverage popularly known as “Brazil nut milk”, belonging to a sector of alternative beverages to dairy milk. In recent years, this sector has shown an increasing in demand, driven by several reasons such as the “negative” effects of cow’s milk in certain consumer groups, e.g., protein allergy, lactose intolerance, the content of saturated fat, and the presence of cholesterol; as well as the preference of people with a healthy diet and lifestyle, those conscious of the environment, and concerns for animal welfare [4]. Although plant-based milk usually has added sugar and is low in protein, its appreciable content of unsaturated fatty acids, essential minerals, phenolic compounds, and other bioactive compounds makes it a healthy food alternative. However, the market for plant-based milk is limited to a few sources (mainly soy and almond), and further study is needed to focus on their bioactive compounds, as well as their effect on processing and the remaining by-products [5]. The scarce reports on Brazil nut beverages are mainly related to standardization (raw material/water ratio, homogenization temperature, formulation, drying, among others) and quality (sensory, nutritional, physicochemical, and microbiological) [6,7,8,9], with an absence of studies related to its bioactive compounds and their biological properties, as well as the potential use of its by-products generated in their processing.

Therefore, the present work seeks to characterize the nutritional attributes, the phytochemicals, and the antioxidant properties of Brazil nuts, its beverage, and by-products, in order to evaluate their potential as healthy food alternatives and healthy ingredients. In the context of sustainable production, this study supports the investigation of the functional beverages of the Brazil nut and the use of its generated by-products.

## 2. Materials and Methods

### 2.1. Plant Material

Brazil nuts, dry seed, and shelled (without woody tegument), were purchased from a local market in Madrid (Spain) in April 2019. The vacuum packaged Brazil nuts were stored in a refrigerated room with an average temperature of 4 °C until analysis and processing. All processing and analyses were carried out over a period of four months.

### 2.2. Solvents, Reagents, and Standards

Methanol (99.8% LC-MS) was purchased from VWR International (Barcelona, Spain). Ultra-pure water (Mili-Q) was obtained from a Millipak^®^ Express 40 system (Merck Millipore, Darmstadt. Germany). The reagents acetone, sodium carbonate, n-hexane, formic acid, and chloroform were purchased from VWR (Barcelona, Spain). Potassium peroxydisulfate (K2S2O8), potassium phosphate (KH_2_PO_4_), sodium phosphate (NaH_2_PO_4_), Trolox (6-hydroxy-2,5,7,8-tetramethylchroman-2-carboxylic acid), fluorescein, N, O-bis (trimethylsilyl) trifluoroacetamide (BSTFA), Folin–Ciocalteu reagent, 2,2-diphenyl-1-picrylhydrazyl (DPPH), 2,20-azino-bis(3-ethylbenzthiazoline-6-sulfonic acid) (ABTS), 2,20-azobis (2-methylpropionamidine) dihydrochloride (AAPH), standards for GC analysis (DL-α-tocopherol, squalene, oleic acid, CI-stearoyl-RAC Glycerol and β-sitosterol), and for HPLC analysis (catechin, epicatechin, vanillic acid, protocatechuic acid, quercetin, vanillin, gallic acid and hydroxybenzoic acid) were purchased from Sigma-Aldrich (Missouri, MO, USA). For protein analysis: Tris-HCl and Trizma base (Sigma-Aldrich), SDS (Merck, Darmstadt, Germany), glycerol (Panreac Química SAU, Castellar del Vallès, Spain), β-mercaptoethanol (Sigma-Aldrich, Madrid, Spain), bromophenol blue (Merck, Darmstadt, Germany), bis-trispolyacrylamide gels (Criterion XT, Bio-Rad, Hercules, CA, USA), XT MES running buffer 20x (Bio-Rad Laboratories S.A, Madrid, Spain), and Coomassie Blue (Instant blue, Expedeon, UK).

### 2.3. Production of Brazil Nut Beverage

**Figure 1:**
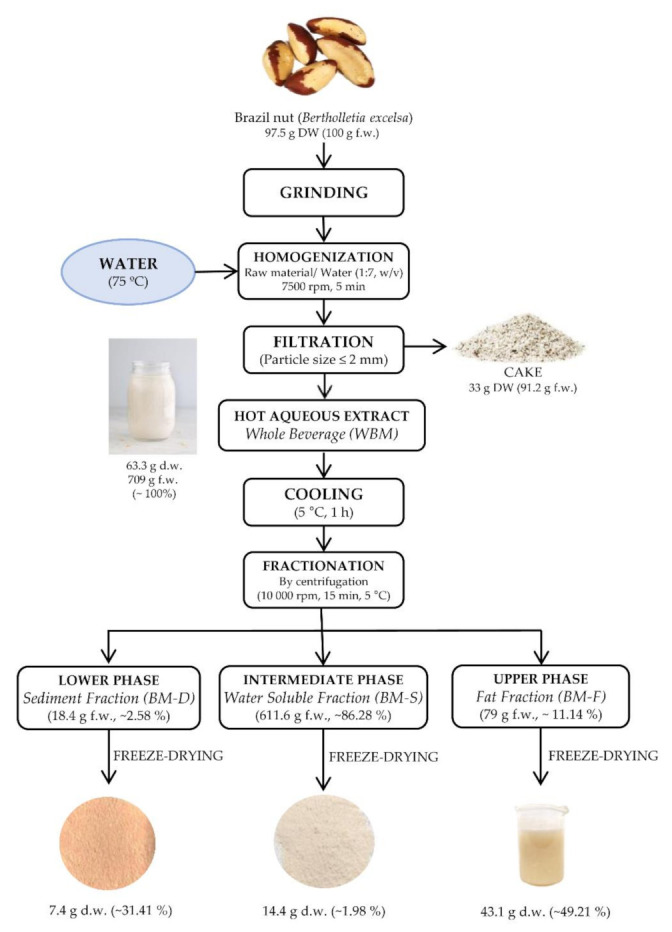
Flow chart of the Brazil nut milk production and process by-products.

The production of whole Brazil nut beverage (WBM) was obtained using the method reported by Felberg et al. as a reference [6]. A centrifugation stage was added for partial fractionation and as a strategy to reduce the level of fat and obtain the water-soluble beverage (BM-S), as shown in Figure 1. Briefly, the Brazil nuts were ground to reduce their size (particle size < 5 mm), then they were homogenized with water at 75 °C using the ratio 7:1 (water: raw material, *v*/*w*) for five minutes until the mixture reached a homogeneous consistency via a high-speed homogenizer at 7500 rpm (OMNI Macro-ES Programmable Homogenizer, OMNI International, Kennesaw, GA 30144, United States). Then, this solution was immediately filtered with stainless mesh (≤2 mm) to obtain a hot aqueous extract or WBM and cake by-product. For the fractionation of WBM, the liquid was maintained at 5 °C for 1 h and then it was centrifuged at 15,000× *g* at 5 °C for 15 min, producing its separation in three phases: the fat fraction (BM-F) from the upper phase, the water-soluble fraction (BM-S) from the intermediate phase, and the sediment fraction (BM-D) from lower phase. Each fraction was manually collected. Finally, all the fractions were frozen with liquid nitrogen, freeze-dried, packed in vacuum bags, and kept at −20 °C until analysis.

### 2.4. Methods of Analysis

#### 2.4.1. Physicochemical Analysis

The centesimal composition of the samples was determined using the following methods: ash, moisture, and dry matter were analyzed following AOAC standardized methods [10]; the total protein content was determined by the elemental chemical analysis of nitrogen (conversion factor: 6.25); the total lipid content was determined by the Folch method [11]; the total carbohydrate was calculated by the difference. Regarding the physicochemical properties, the pH was determined by direct reading on a digital potentiometer (Metrohm 827 pH Meter, Metrohm, Herisau, Switzerland), the total acidity concentration in the samples was calculated via potentiometric titration [10], and the °Brix (soluble sugars) was measured using a digital refractometer (PR-32α, ATAGO™, Tokyo, Japan).

#### 2.4.2. Phenolic Compounds

##### Extraction

Phenolic compounds were extracted using the method reported by John and Shahidi, with some modifications [12]. Each sample was previously defatted with hexane (1:5 *w*/*v*) and homogenized for 5 min, in triplicate. The obtained solution was then filtered through filter paper. The solid residue (defatted) was dried at room temperature for 12 h in the dark and covered to avoid oxidation. It was then extracted with acetone, in a ratio of 1:15 (*w*/*v*), homogenized at 7500 rpm for 4 min in an ultra-speed homogenizer (OMNI Macro-ES Programmable Homogenizer, OMNI International, Kennesaw, GA, USA), and later submitted to reflux in a water bath at 60 °C for 40 min with magnetic stirring. The resulting slurry was centrifuged at 15,000× *g* for 10 min, and the supernatant was collected. The sediment was re-extracted under the same conditions, then the supernatants were combined, and the solvent was removed by vacuum at 40 °C. The extract containing the phenolic compounds was stored at −20 °C until its use for the analysis of the total phenolics and antioxidant activity. For the HPLC analysis of the individual phenolic compounds, the extract was freeze-dried (Lyobeta-15, Azbil Telstar, S.L., Terrasa, Spain) for 72 h and the samples were stored at −20 °C until analysis.

##### Total phenolics Content (TPC)

The total phenolics in the samples (phenolic extracts) were analyzed spectrophotometrically with Folin–Ciocalteu reagent according to the method of Cano et al. [13], with some modifications. In a 96-microwell plate, the reactants were placed in the following order: 20 μL samples (extracts, standard, and blank) were reacted with 100 μL Folin–Ciocalteu reagent (10%, *v*/*v*) and then alkalized with 80 μL of Na_2_CO_3_ 7.5% (*w*/*v*). The mixture was stirred and kept in darkness for one hour at 20 °C. The reading was taken spectrophotometrically at 756 nm (Varioskan Flash, Thermo Fisher Scientific, Waltham, MA, USA). Gallic acid was used as the reference standard to elaborate the calibration curve with concentrations in the range of 50–500 μg/mL. The results were expressed as mg of gallic acid equivalents (GAE)/100 g dry weight (d.w.) or fresh weight (f.w.).

##### Individual Phenolic Compounds

Phenolic lyophilized extracts (BN, BM-S, and BM-D) were dissolved with methanol/water (2:1, *v*/*v*) and filtered with 0.45 µm syringe filters before injection into HPLC UV/Vis. Chromatographic analyses were performed according to the protocol reported by García-Cayuela et al. [14], using a 1200 Series Agilent HPLC System (Agilent Technologies, Santa Clara, CA, USA) with a reverse phase C18 column (Zorbax SB-C18, 250 × 4.6 mm, 5 μm; Agilent) maintained at 20 °C. Elution solvent A consisted of 1% formic acid (*v*/*v*) in water, while solvent B was a mixture of methanol and formic acid (1%, *v*/*v*). Separation was achieved using an initial solvent composition of 15% (B) during 15 min, increased to 25% (B) within 10 min, ramped to 50% (B) within 10 min, increased to 75% (B) in 15 min, and followed by a decreased period of 15% (B) in 5 min prior to isocratic re-equilibration at 15% (B) for 10 min. The flow rate was fixed at 0.8 mL/min, and the injection volume was 20 μL. The UV–vis photodiode array detector was set at 3 wavelengths (280, 320, 380 nm) for monitoring different phenolic chemical families simultaneously.

The HPLC-DAD was coupled to a mass spectrometry detector (LCMS SQ 6120, Agilent, CA, USA) with an electrospray ionization (ESI) source operating in positive ion mode. The drying gas was nitrogen at 3 L/min at 137.9 KPa. The nebulizer temperature was 300 °C, and the capillary had 3500 V potential. The coliseum gas was helium, and the fragmentation amplitude was 70 V. Spectra, recorded *m*/*z* from 100–1000. Further mass spectrometry analyses were performed using maXis II LC-QTOF equipment (Bruker Daltonics, Bremen, Germany) with an ESI source and the same chromatographic conditions. The ESI-QTOF detector worked in positive ion mode and recorded spectra *m*/*z* from 50–3000. The operation conditions were 300 °C, capillary voltage 3500 V, charging voltage 2000 V, nebulizer 2.0 bar, and dry gas at 6 L/min. MS/MS analysis used the bbCID (broadband Collision-induced dissociation) method at 30 eV.

Phenolic compounds were identified according to their retention times, UV/Vis, and mass spectral data compared to those of commercial or purified standards. Quantitation of most phenolic compounds was determined using the calibration curves of the corresponding isolated standards. Quercetin glycosides were quantified by using the rutin calibration curve (Figures S1 and S2).

#### 2.4.3. Lipids and Other Minor Lipidic Compounds

##### Extraction

The lipids in the samples were extracted using the method of Folch, Lees, and Sloane Stanley [11] as follows. Briefly, 1 g of sample was mixed with 20 mL of chloroform-methanol (2:1, *v*/*v*) to be homogenized in a T-25 Ultraturrax (IKA-Werke GmbH & Co., KG, Staufen, Germany) at 11,000 rpm for 3 min, followed by sonication by ultrasound bath for 3 min and then centrifuged at 1050× *g* for 10 min. The supernatant was collected and stirred with 4 mL of distilled water by vortex and then centrifuged again at 1050× *g* for 10 min. The bottom organic phase was collected and filtered with paper and dried with anhydrous sodium sulphate. The lipid solution obtained was dried on a rotary evaporator under a vacuum at 40 °C and kept at −20 °C until analysis.

##### Fatty Acids

The fatty acids (FA) profile of lipid samples was determined after the derivatization of fatty acid methyl esters (FAMEs). Briefly, 20 mg of sample was mixed with 0.5 mL chloroform/methanol (2:1; *v*/*v*) and 1 mL of 0.1 M NaOH in methanol. This mixture was heated at 60 °C for 30 min, and then the reaction was stopped with the addition of 0.2 mL of distilled water. The FAMEs formed were extracted with the addition of 1 mL of hexane. After stirring, it was decanted, and the isolated upper phase was collected. Anhydrous sodium sulfate was added to the solution of FAMEs to eliminate aqueous traces for 30 min. Finally, the hexane was removed under a nitrogen atmosphere.

The obtained FAMEs were dissolved in hexane to a concentration of 1 mg/mL for each sample and injected into the gas chromatograph Agilent 7890 A (Agilent Technologies, California, PA, USA) equipped with a split-splitless injector and an autosampler, a flame ionization detector, and a triple-axis mass spectrometer detector. FAMEs were separated using an HP-5MS column (30 m length, 0.25 mm internal diameter, and 0.25 µm film thickness). Helium was used as a carrier gas at 2 mL/min. The injection temperature was 260 °C, splitless mode, 1 µL, and the mass spectrometer ion source and interface temperatures were 230 and 280 °C, respectively. The chromatographic analysis started at 50 °C and increased at a speed of 20 °C/min until it reached 210 °C, there it stayed for 18 min, then it went up to 230 °C and remained there for 13 min. The running time was 40 min. The mass spectra were obtained by electronic impact at 70 eV. The scan rate was 1.6 scans/s at a mass range of 30–700 amu. The identification of fatty acids was performed by the NIST MS Data library. The area under each FA peak in relation to the total area of all FA peaks was used for quantification and expressed as a percentage of FA.

##### Minor Lipid Compounds: Free Fatty Acids, Monoglycerides, Tocopherols, Phytosterol, and Squalene

For the analysis of these minor compounds, the lipid extracts were derivatized using N, O-bis(trimethylsilyl)trifluoroacetamide (BSTFA). The procedure used was based on that of Herrera et al. [15]. Briefly, 5 mg of the sample was mixed with 1 mL of the derivatizing agent and then submitted to 75 °C for 1 h, shaking every 15 min. Finally, it was injected into the previously described chromatography equipment, and the separation procedure was started at 50 °C and was held for 3 min, increasing to 310 °C at a rate of 15 °C/min and held for 25 min. For quantification, calibration curves were obtained from the standards of α-tocopherol, squalene, oleic acid, stearoyl-RAC glycerol, and β-sitosterol, which were also previously derivatized.

#### 2.4.4. Proteins

##### SDS-PAGE Analysis

The electrophoresis assay was performed on Precast Criterion XT 4–12% Bis-Tris gels using the Criterion cell (Bio-Rad, Hercules, CA, USA) as reported by Sanchón et al. [16]. The samples were dissolved at 0.8 mg of protein/mL in a buffer containing 0.05 M Tris-HCl, pH 6.8, 8% (*v*/*v*) glycerol, 1.6% (*w*/*v*) SDS, 2% (*v*/*v*) β-mercaptoethanol, and 0.002% (*w*/*v*) bromophenol blue. The concentration was calculated at 0.8 mg protein/mL buffer solution. Then, they were heated at 95 °C for 4 min, and 45 μL was loaded onto the 4–12% Bis-Tris polyacrylamide gel as well as 15 μL of the molecular weight marker (Precision Plus Protein unstained Standard (Bio-Rad, Bio-Rad, Hercules CA, USA). Electrophoretic separation was carried out using XT-MES as a running buffer (BioRad). After 5 min at 100, 150 V were applied. The gels were washed with Milli-Q water and were stained with Coomassie blue G-250. Images were taken with a Molecular Imager^®^ VersaDoc™ MP 5000 system (Bio-Rad, Hercules, CA, USA) and processed with Quantity One^®^1-D analysis software (Bio-Rad Laboratories S.A, Madrid, Spain).

##### Mass Spectrometry Analysis of Peptides

To identify the proteins on the SDS-PAGE gels, bands were cut out from the gels and transferred to a microcentrifuge tube. Subsequently, in-gel reduction, alkylation, and tryptic digestion were carried out as previously reported by Miralles et al. [17]. An Autoflex speed MALDI-TOF/TOF (Bruker Daltonics, Bremen, Germany) instrument was used for mass spectra generation. The Mascot server (www.matrixscience.com, Matrix Science, London, UK) was used to carry out protein identification searches against a homemade database of *Bertholletia excelsa* and *Pisum sativum* proteins selected from the UniProt database (https://www.uniprot.org/ (accessed on 22 May 2020)).

#### 2.4.5. Minerals

##### Selenium

For the quantification of selenium (Se), the protocol proposed by López-Bellido et al. [18] was followed, with some modifications. Briefly, the dry samples (approximately 0.5 g) were pretreated by acid digestion, using HNO_3_ in a microwave (UltraWAVE Milestone, Shelton, CT, USA). Then, the samples were diluted 1:2 (*w*/*v*) with 1% nitric acid and analyzed by inductively coupled plasma mass spectrometry (ICP-MS model NexION 300 X, Perkin Elmer^®^, Waltham, MA, USA) under the following operating conditions: nebulizer quartz concentric, spray chamber PC3 Peltier Cooled Cyclonic, RF Power 1500 W, nebulizer gas flow of 0.86 L/min, scanning mode in peak hopping, d.w.ell time of 50 ms, 20 sweeps, 1 reading, 3 replicates, analytes ^78^Se and ^80^Se, internal standard ^72^Ge, and reaction mode CH4 = 0.70 mL/min and RPq = 0.55. Calibration standards at 0.05, 0.1, 0.5, 1, 5, 10, and 50 μg/L were prepared from a 1000 mg/L stock solution of Se (SCP Science) in 1% nitric acid. One internal standard (Ge at 30 μg/L) was used to correct for signal drift. The results were expressed as µg/100 g d.w. or f.w.

##### Other Minerals

The analysis was performed by the high-resolution atomic absorption spectrometry (HR-CS AAS) method according to the procedure suggested by Nielsen [19], with certain modifications. First, the samples were digested: 1 g of the samples, with nitric acid and hydrogen peroxide (both of high purity), were added and submitted to a programmed temperature of 200 °C using the microwave digestion system equipment (Ethos UP, Milestone Srl, Sorisole, Italy). The digested samples were diluted with 50 mL of distilled water. In the second stage, the HR- CS AAS was used (ContrAA 700, Analytik Jena AG, Jena, Germany). Air-acetylene flame was applied for all minerals except calcium (Ca) and phosphorus (F). The Ca required the more calorific acetylene nitrous oxide flame. F was determined using a graphite furnace. Each element was determined by the previous calibration of specific certified standards. The minerals obtained were expressed in mg/100 g d.w. or f.w.

#### 2.4.6. Antioxidant Capacity

##### DPPH Assay

The antioxidant capacity was determined by the DPPH (2,2-diphenyl-1-picrylhydrazyl) radical scavenging method according to the method of Abe et al. [20], with some modifications. In a 96-well microplate, the reaction was carried out as follows: a 50 µL aliquot of the sample extract, previously diluted with methanolic solution (70%), was mixed with 250 µL of DPPH (0.5 mM) and after 25 min in the dark, the absorbance was measured at 517 nm using spectrophotometric equipment (Varioskan Flash de Thermo Electron Corporation^®^, Waltham, MA, USA) and analyzed with SkanIt Re for Varioskan 2.4.1^®^ software (Varioskan Flash de Thermo Electron Corporation^®^, Waltham, MA, USA). The reference standard for obtaining the calibration curve consisted of a methanolic solution of Trolox (6-hydroxy-2,5,7,8-tetramethylchroman-2-carboxylic acid) at different concentrations (100–800 µM/mL). The antioxidant capacity was expressed as µmol Trolox equivalent (TE)/g d.w. or f.w.

##### TEAC Assay

The radicals ABTS+ were generated according to Koroleva et al. [21], with some modifications. ABTS and potassium peroxodisulfate (K2S2O8) were dissolved in water at final concentrations of 7 and 140 mM, respectively. The ABTS stock solution was obtained by mixing both reagents in a ratio of 17.6 µL K2S2O8/mL ABTS. To get a daily solution of radical ABTS, the stock solution was diluted with phosphate-buffered saline (PBS, 75 mM, pH 7.4), constituted by potassium phosphate (KH2PO4) and sodium phosphate (NaH2PO4), until an optical density of 0.70 ± 0.02 at 734 nm. TE solutions (10–100 µM) were used for calibration. The reaction was initiated by mixing 30 µL of the sample extract, or standard, with 200 µL of ABTS+, then left to stand 40 min and the absorbance was measured spectrophotometrically at 734 nm. Values were calculated based on the linear regression equation between the TE concentration and the decrease in absorbance. Antioxidant capacity was expressed as µmol TE/g d.w. or f.w.

##### ORAC Assay

The oxygen radical antioxidant capacity (ORAC) was determined according to the method proposed by Gómez-Maqueo et al. [22], with some modifications. The extracted samples and TE were dissolved with phosphate-buffered saline (PBS) 75 mM at pH 7.4. A TE curve was prepared using concentrations ranging from 10–45 mM. In a microwell plate, 20 mL of the sample extract, or standard, was added to each microwell, then 120 mL of 11.7 µM fluorescein solution was added. The microplate was incubated at 37 °C for 10 min. Afterwards, 60 mL of a 153 mM 2,2′-Azobis (2-methylpropionamidine) dihydrochloride (AAPH) solution were added. In a 96-well microplate reader (Varioskan Flash de Thermo Electron Corporation^®^, Waltham, MA, USA), plate lecture was registered every minute for 55 min at 37 °C and with an excitation wave of 485 nm and emission wave of 530 nm. The data analysis was performed by obtaining the area under the curve (AUC) and subtracting the blank. The results were expressed as μmol TE/g d.w. or f.w.

#### 2.4.7. Statistical Analysis

The values are expressed as mean and standard deviation. The obtained results were evaluated with variance analysis (ANOVA), and differences between means were located using Tukey’s test. The significant statistical differences were calculated at a *p* < 0.05 level. The statistic software employed was IBM SPSS Statistic 20.

## 3. Results and Discussion

### 3.1. Production of Brazil Nut Beverage

Figure 1 show the production procedure reported in the Materials and Methods section, using a ratio of BN/water 1: 7 (*w*/*v*). In this process, 100 g f.w. of BN with 700 mL of water, 709 g f.w. of WBM and 611.6 g f.w. of BM-S, were obtained. That is, to obtain WBM and BM-S beverage, 14.11 and 16.4% (*w*/*v*) of raw material were required, respectively. In commercial plant-based milks, the raw material content varies in the range of 2–15% (data from field survey, taken from the labels of the main brands of vegetal milk available in Madrid, Spain.) (*w*/*v*), depending on the type of raw material, raw material/water ratio, technological processes, and the prefixed characteristics of the final product. In the literature regarding the production of Brazil nut beverages, the use of various BN/water ratios have been reported, as 1:2 (*w*/*v*) [9] and 1:7 (*w*/*v*) [6]. The latter ratio was used in our work due to the good yield of total solids at 77 °C and the remarkable nutritional profile. A remarkable aspect of BM-S was a better appearance of stability than WBM since fat and sediment fraction was separated by centrifugation. It is true that this process would reduce the formation of sedimentation (particles with higher density), and cream formation (oily bodies of low density) during its storage, but does not affect the nutritional composition, as verified in the following sections.

Regarding the by-products in the process of BN milk production, the cake is the first residue from processing, with an amount of roughly 33 g d.w. from 100 g f.w. (~97.5 g d.w.) produced from the initial raw material (BN). These results are similar to those reported (36.14%, *w*/*w*, d.w.) in a previous study using partially defatted BN to obtain the BN beverage [9], an important amount of food material for subsequent utilization. Furthermore, on a fresh weigh basis, BM-D and BM-F residues represent less than 14% (*w*/*w*) of the WBM, but on a dry basis they are very important and reach 31.4% (*w*/*w*) of BM-D and 49.2% (*w*/*w*) of BM-F, significative in large production volumes.

Regarding the by-products in the process of BN milk production, the cake is the first residue from processing, with an amount of roughly 33 g d.w. from 100 g f.w. (~97.5 g d.w.) produced from the initial raw material (BN). These results are similar to those reported (36.14%, *w*/*w*, d.w.) in a previous study using partially defatted BN to obtain the BN beverage [9], an important amount of food material for subsequent utilization. Furthermore, on a fresh weigh basis, BM-D and BM-F residues represent less than 14% (*w*/*w*) of the WBM, but on a dry basis they are very important and reach 31.4% (*w*/*w*) of BM-D and 49.2% (*w*/*w*) of BM-F, significative in large production volumes.

### 3.2. Physicochemical Properties

The centesimal composition of the Brazil nut, its beverages (BWM and BM-S), and by-products (cake, BM-D and BM-F), are shown in Table 1. The macronutrients of BN, in order of predominance, are the lipids (58.5/100 g f.w.), followed by carbohydrates (19.6/100 g f.w.), proteins (16/100 g f.w.), and ash (3.3/100 g f.w.). The high caloric power of 669.2 Kcal, mainly provided by lipid level, makes BN an energetic food with a valuable nutritional contribution.

In BN beverages, the total solids content of the WBM beverage was about 9/100 g f.w., a higher level than BM-S with around 2/100 g f.w., when the vegetable milk commercials can vary in a range of 3–23% (*w*/*w*) [5]. The nutrients that conform to the WBM were dominated by total lipids with 5.24/100 g f.w., followed by carbohydrates with 1.94/100 g f.w., proteins with 1.43/100 g f.w., ash with 0.33/100 g f.w., and an energy content of 60.6 kcal/100 g f.w. These values are close to those obtained by Felberg et al. [6], who used a similar process.

In the case of the BM-S beverage, it had a higher predominance of proteins (0.8/100 g f.w.) and carbohydrates (0.83/100 g f.w.), and a minimum composition of lipids (0.25/100 g f.w.), ash (0.1/100 g f.w.), and a minimum energy content (8.8 kcal/100 g f.w.). In general, there was a reduction of macronutrients in BM-S compared to WBM (mainly in lipid content, which decreased up to roughly 20 times) due to the centrifugation process and separation of the BM-D and BM-F fractions. Commercial vegetable milks have a wide compositional variation: in protein from 0.1–3.3% (*w*/*v*), in lipids from 0.3–7.2% (*w*/*v*), in carbohydrates from 0–13.4% (*w*/*v*), and in energy content from 21–121 kcal/100 mL [23]. Therefore, WBM and BM-S are beverages comparable to commercial vegetable milk, but the WBM has a high energy input, as opposed to the BM-S.

Regarding the by-products, the cake had a notable nutritional composition of lipids (44.2/100 g d.w.), carbohydrates (35.45/100 g d.w.), proteins (16.85/100 g d.w.), and ash (3.5/100 g d.w.). These values are consistent with those reported by Sartori et al. [9], who produced the beverage from partially defatted BN. In addition, regarding the by-products obtained after centrifugation, the BM-D fraction showed an important content (about 57/100 g d.w.), probably due in part to the effect of the thermal denaturation of some proteins and the increased hydrophobicity [24]. This fact was observed in soy and almond milk when they were subjected to moderate heat treatments (around 60–80 °C), showing lower precipitation and protein denaturation than those produced when they were treated at high temperatures (over 85 °C) [25,26]. In the case of the BM-F fraction, it had a predominance of lipid content (about 80/100 g d.w.), being related to the high-fat content coming from the raw material.

In the physicochemical characteristics (Table 1), the pH ~6.5 of the Brazil nut and cake were consistent with the previously published studies [27]. The pH of WBM and BM-S were within the range (pH: 5–8) of most vegetable milks [5]. The high level of acidity (0.54 g citric acid/100 g) of the sediment (BM-D) could be influenced by amino acids due to their high protein content (~57%, *w*/*w*). Regarding the content of soluble solids, the BN has the highest value with 14.5 °Brix due to low moisture (2.5%, *w*/*w*), and the WBM and BM-S have ~3 °Brix, due to dilution in water of BN processing. The by-products (BM-D and cake), in dry weight, have a low content of soluble solids that can be attributed to the effect of the hydrothermal process for obtaining the beverage and the consequent extraction of water-soluble substances for BN.

### 3.3. Phenolic Compounds

#### 3.3.1. Total Phenolic Content (TPC)

The Folin-Ciocalteau method allowed the quantification of TPC present in the samples. Table 2 show these data. The TPC of BN was 108 mg GAE/100 g d.w. (~100.1 mg GAE/100 g f.w.), this value was similar to a previous report [20] but differed from that obtained by John and Shahidi [12], who reported 519 mg GAE/100 g f.w. This difference can be attributed to different factors inherent to the plant, industrial processes, and methods of extraction and analysis.

Regarding the Brazil nut beverages, the WBM and BM-S showed similar total phenol content, with 7.1 and 6.2 mg GAE/100 g f.w., respectively. These were higher values than those reported for commercial vegetable milks, which ranged from 0.02–1.24 mg/100 mL [28]. However, some studies may show higher phenolic concentrations, such as almond milk with 71 mg/100 g f.w. [29].

When analyzing the TPC level on a dry basis, according to Table 2, the BM-S (314 mg GAE/100 g d.w.) was significantly higher (*p* ≤ 0.05) than the rest of the samples (<84 mg GAE/100 g d.w.). According to the data of John and Shahidi [12], the phenolic extract of BN contained roughly 97% (*w*/*w*, fresh weight) soluble phenolics and less than 5% (*w*/*w*, fresh weight) bound phenolics, this would explain the higher TPC in the BM-S beverage.

Regarding the cake by-product, with 49.2 mg GAE/100 g d.w., it has a TPC nearly to half of BN, a reduction consistent with the high concentration of soluble polyphenols present in the BN transferred to the aqueous fraction (BM-S). There are no reports of TPC in cake from the BN beverage. However, Gomes et al. [30], in their study of phenolic extraction of cake (from defatting of the BN), obtained a TPC from 143–182 mg GAE/100 g. In the cake (by-product) of soybean milk, known as “okara”, the total phenol content is recorded to be from 620–2913 mg GAE/100 g d.w. [31,32]. Therefore, at the level of total phenols, the cake from the BN beverage process is relatively low. As for the BM-D fraction, precipitated by-product, with 83.4 mg GAE/100 g d.w., their total phenols would be to a greater extent conjugated (esterified) with amino acids and peptides (due to their high total protein content ~57% in dry weight), decanted by factors such as higher molecular weight, lower aqueous solubility (as globulins and glutelins), and protein denaturation. The fat fraction (BM-F) was not analyzed for total phenol content due to its high-fat content (greater than 80%, *w*/*w*, dry weight) and the antecedents regarding its minimum TPC level of BN oil, as 3.6 mg GAE/100 g oil [33].

#### 3.3.2. Identification of Individual Soluble Phenolic Compounds

**Figure 2:**
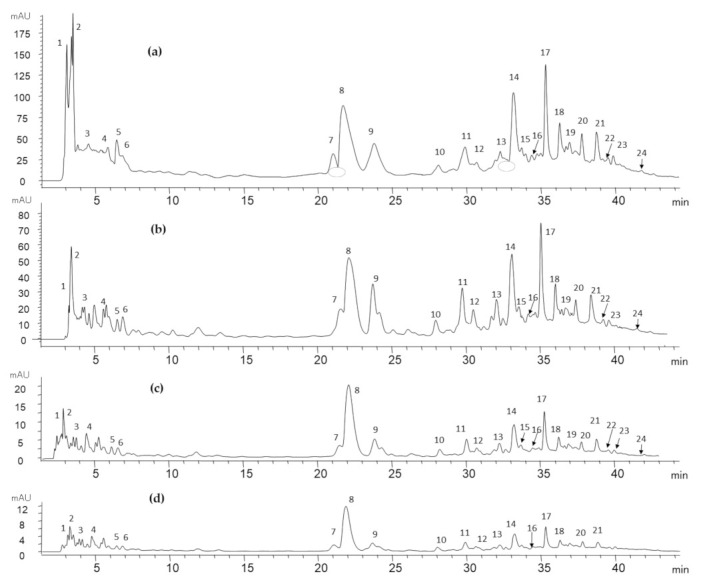
HPLC C18 chromatogram of phenolic compounds from (**a**) BM-S, (**b**) Brazil nut (*Bertholletia excelsa* HBK), (**c**) cake and (**d**) BM-D at 280 nm.

After the extractions, in an aqueous solvent (70% acetone), the soluble phenols of each sample were characterized. The profile of phenolic compounds of the Brazil nut, water-soluble fraction (BM-S), and sediment fraction (BM-D) was conducted by HPLC-DAD (UV/Vis detection) and by HPLC- ESI-QTOF, analyzing retention times, UV spectra, mass spectral data compared to those of commercial and purified standards, and to reported data (Table 3). The UV-vis detection was simultaneously recorded at 280, 320, and 380 nm, achieving the identification of 24 compounds: 4 organic acids, 10 phenolic acids, 9 flavonoids, and 1 phenolic aldehyde. The HPLC chromatogram at 280 nm (Figure 2) showed a better resolution of the peaks for the identification of the phenolic compounds present in the samples. Table 3 show the HPLC retention times, UV/Vis spectra, and MS spectral data of individual phenolics in the Brazil nut (*Bertholletia excelsa*), water-soluble fractions (BM-S), and sediment fraction (BM-D).

Peaks detected in the first five minutes of the run were identified as organic acids. Those were citric acid (peak 1, Rt = 2.9 min), ascorbic acid (peak 3, Rt = 3.8 min), and succinic acid (peak 4, Rt = 4.8 min), distinguished by their common pattern of fragment ions and agreement with previous reports [12] and other fruits [34,35]. Regarding peak 2 (Rt = 3.59 min), its precursor ion *m*/*z* 87 was identified as pyruvic acid [36]. The phenolic acids identified were mostly hydroxybenzoic acids (eight compounds in their free or derivatives form of gallic acid, ellagic acid, 4-hydroxybenzoic acid, protocatechuic acid, and vanillic acid) and, to a minor degree, by hydroxycinnamic acids (ferulic acid, and p-coumaric acid) and phenolic aldehyde (vanillin). Peak 5 (Rt = 6.4 min) corresponded to gallic acid with a precursor ion *m*/*z* 274 and a daughter ion at *m*/*z* 125, a match with previous studies of the Brazil nut [12]. Peak 6 (Rt = 6.9 min), due to the fragment, formed *m*/*z* 169, the compound was identified as a derivative of gallic acid [12]. Peak 7 (Rt = 21.7 min) was identified as protocatechuic acid derivative, due to its pattern of fragmentation *m*/*z* 153, 159, and 124, and in agreement with the values reported in the Brazil nut by John and Shahidi [12], who found a higher abundance of protocatechuic acid and its derivative in the BN skin. Peak 10 (Rt = 28.4 min) had an important signal and was identified as 4-hydroxybenzoic acid, confirmed by the UV spectrum of its standard and similarity of molecular ion [M-H]^−^ *m*/*z* 137 observed in almond, as previously reported [38]. In peak 11, the UV detected spectrum (Rt = 30.2 min) corresponded to vanillic acid, with molecular ion *m*/*z* 167, which at the same time is a product ion peak of 24 (Rt = 42.1 min), identified as vanillic acid derivative, coinciding in a previous report [12,38]. Peak 13 (Rt = 32.4 min) was tentatively identified as vanillin with a precursor ion *m*/*z* 151; although not commonly reported in nuts, the precursor ion was also found in wheat [40]. Peak 17 (Rt = 36.28 min) corresponded to the molecular ion [M-H]- *m*/*z* 164, indicative of p-coumaric acid, with product ion *m*/*z* 119, with fragment denominates, such as decarboxylated coumaric acid in walnut, according to Pycia et al. [39]. Peak 18 (Rt = 36.4 min), a compound with a high signal, has a similr UV spectrum to ferulic acid (λmax 230 nm) and matched to the fragment ion *m*/*z* 133 reported in walnuts [39]. Peak 20 (Rt = 37.9 min) showed a fragmentation pattern which corresponded to an ellagic acid derivative, conformed to ion *m*/*z* 301 (corresponding to ellagic acid) found at peak 22 (Rt = 39.7 min), with values consistent to the previous report on the Brazil nut [12].

**Table 3:** HPLC retention times, UV/Vis spectra and MS spectral data of individual phenolics in the Brazil nut (*Bertholletia excelsa* HBK), BM-S, BM-D, and cake.

Peak ^1^	Rt (min)	Assigned Identity	UV λ_max_ (nm)	[M-H]^−^ *m*/*z*	MS/MS *m*/*z*	References
1	2.9	Citric acid ^2^	209	191	111, 173	[12,34]
2	3.1	Pyruvic acid ^2^	227.8 (259.7)	87.06	59.01	[36]
3	3.8	Ascorbic acid ^2^	289	175.03	147.2, 87.00, 69.03	[34]
4	4.8	Succinic acid ^2^	228	117.02	72.91	[35]
5	6.4	Gallic acid	274	169.01	125.02, 107.01, 97.03, 79.02, 69.03, 51.02, 41.04	[37]
6	6.9	Gallic acid derivative	272	187	125.02, 169.01	[12]
7	21.7	Protocatechuic acid derivative	280	−	153.04, 109.03, 124.03	[12]
8	22.3	Catechin	230, 280	289.1	136.8, 150.7, 160.8	[12,38]
9	24.0	Catechin derivative	282	−	289.1	[12]
10	28.4	4-hydroxybenzoic acid	252	137.03	106.64, 93.03	[38]
11	30.2	Vanillic acid	259, 292	167.03	152.01, 108.02	[12,39]
12	30.9	Epicatechin	279	289.1	109.01, 121.01, 123.03, 125.01, 137.00	[38]
13	32.4	Vanillin	274, 309	151.05	137.05, 123.05, 109.0, 81.0	[40]
14	33.4	Catechin gallate	231.8, 280.1, (324.8)	441.03	109.01, 125.00, 168.98, 289.03	[41,42]
15	33.8	Epicatechin gallate	232.6, 280 (324.8)	441.19	109.08, 125.08,137.08, 151.10, 203.14, 245.16, 289.16	[38]
16	34.6	Epigallocatechin 3-O gallate	232; 280 (312)	457.3	305.6, 169.1, 125.02	[43]
17	35.4	p-Coumaric acid	227, 310	164.05	119.05, 91.05	[39]
18	36.4	Ferulic acid	(292), 323	193.1	177.1, 161, 133.1	[39]
19	37.0	Taxifolin (dihydroquercetin)	231.9, (282.8) 309.2	303.05	285.05, 179.00, 125.03	[12]
20	37.9	Ellagic acid derivative	252, 360	447	301, 257, 229	[12]
21	38.9	Quercetin	232, 323	301	179, 151	[12,44]
22	39.7	Ellagic acid	254, 368	301	285, 283, 257, 229, 184.92, 134.92	[12]
23	40.0	Myricetin-3-O-rhamnoside	253, 370	463	317	[12,45]
24	42.1	Vanillic acid derivative	259, 294	329	167	[12]

^1^ Peak numbers correspond to those of Figure 2; ^2^ Organic acids.

In the flavonoid group of the Brazil nut and derivatives samples, six types of flavan-3-ols were detected (mainly catechin, epicatechin, and their gallate ester or derivatives), followed by two flavonols (myricetin-3-O-rhamnoside and quercetin), and one flavanonols (taxifolin). Peak 8 (Rt = 22.3 min), peak 9 (Rt = 24 min) and peak 14 (Rt = 33.4 min) were identified as catechin, catechin derivative, and catechin-gallate, respectively. The catechin had the highest signal in flavonoids and its identification was confirmed by the UV spectrum of its standard and pattern ion *m*/*z* 289, a common value reported in the Brazil nut and walnut [12,38].

Peak 12 (Rt = 30.9 min) and peak 15 (Rt = 33.8 min) showed a precursor ion *m*/*z* 289 and 441, that corresponds to epicatechin and epicatechin gallate, respectively, which was also observed in almond [38]. Peak 16 (Rt = 34.6 min) with molecular ion [M-H]^−^ *m*/*z* 457 was tentatively identified as epigallocatechin 3-O gallate, which exhibited the loss of a typical fragment ion *m*/*z* 169 that corresponds to gallic acid. Peak 19 (Rt = 37 min) was identified as taxifolin (dihydroquercetin), due to the common pattern ion *m*/*z* 303 and fragmentation pattern *m*/*z* 285 and 125, values that were also observed in a previous report on the Brazil nut [12]. Quercetin was clearly recognized in peak 21 (Rt = 38.9 min) by its principal ion *m*/*z* 301 and fragmentation pattern *m*/*z* 179 and 151, consistent with reports of walnut [44]. Myricetin-3-O-rhamnoside was identified in peak 23 (Rt = 40 min) with [M-H ]^−^ *m*/*z* 463, also observed in reports on the Brazil nut and hazelnut [12,45]. In general, the phenolic characterization of BN is scarce in the literature. We found individual phenols that were not detected or analyzed in previous reports, such as vanillin, catechin gallate, epicatechin, epicatechin gallate, epigallocatechin 3-O gallate, and myricetin-3-O-rhamnoside.

#### 3.3.3. Individual Phenolic Compounds Content in BN and Derivates

The quantification of phenolic compounds in the Brazil nut, WBM, BM-S, and BM-D, are shown in Table 4. The BN had a total phenolic content of 575 µg/g f.w., composed of flavonoids (393.4 µg/g f.w.), phenolic acids (176.1 µg/g f.w.), and vanillin (5.6 µg/g f.w.), which is a phenolic aldehyde. This phenolic composition is common in other nuts, showing a predominance of flavonoids and phenolic acids [46].

According to Table 4, the predominant flavonoids of BN, in free or derivative form, were catechin and epicatechin, and at a lower level of concentration were quercetin, taxifolin, and myricetin-3-O-rhamnoside. These compounds belong mainly to flavan-3-ols and flavonols subgroups. This profile is consistent with the general characteristics of the nuts, where the flavan-3-ols, flavonols, and anthocyanins are considered to be the main flavonoids [47]. Moreover, previous studies also consider catechin and its derivatives as one of the main flavonoids present in BN [30,48]. With respect to the phenolic acids of BN, the most abundant phenolics were mainly hydroxybenzoic acids, free and its derivatives, among them ellagic acid, protocatechuic acid, and vanillic acid. The results are consistent with previous reports of BN, indicating the predominance of gallic acid and protocatechuic acid [12,30,48]. In addition, the present study agreed with those reported for other nuts comprised mainly of hydroxybenzoic acids. For example, in walnuts, pecans, and pistachios, gallic acid and its derivatives were the most abundant; in hazelnuts, the gallic acid and the protocatechuic acid were most prevalent; and in almonds, protocatechuic, vanillic, and p-hydroxybenzoic acid dominated [46]. Considering the above, the phenolic profile obtained from the BN showed several common aspects with previous studies, but also noted distinctions that contribute to the characterization.

Regarding the beverages obtained from Brazil nuts, according to Table 4, the WBM and BM-S had a similar total phenol content of 60.2 and 53.3 µg/g f.w., respectively. In both beverage samples, flavonoids, such as catechin, epicatechin, and their derivatives, stand out, but an inverse fact was seen in BM-D and cake by-products, where phenolic acids are more abundant than flavonoids (Table 4). These results suggested that most of the flavonoids in BN are water-soluble due to the effect of beverage processing and are transferred to the aqueous phase, but their presence is reduced in nonwater-soluble matrices such as BM-D and cake. This fact agrees with the report of John and Shahidi [12], who noted that phenolic acids, such as protocatechuic acid, gallic acid, and ellagic acid, are mainly concentrated in the BN skin, which was the non-water-soluble matrix found primarily in the BM-D. In the literature, reports on the phenolic profile of vegetable milks are scarce, with the exception of soy milk, which showed a higher level of phenolic content than that observed for Brazil nut beverages studied here (WBM and BM-S). In soy milk, the level of flavonoids, such as isoflavonoids, are very important and may vary according to the variety of soy legume [49].

The phenolic compounds identified as predominant in BN and its derivatives, such as flavan-3-ols (catechin and epicatechin) and hydroxybenzoic acids (ellagic acid, vanillic acid, and protocatechuic acid), are widely recognized in the literature with promising effects associated with human health promotion and the prevention of disease risk by acting as an antioxidant, anti-inflammatory, cardioprotective, and antimicrobial, among others [50,51]. Therefore, Brazil nuts (BN), beverages of BN (WBM and BM-S), and its by-products, represent a contribution to the diet through their phenolic phytochemicals.

### 3.4. Lipids and Other Lipophilic Components

#### 3.4.1. Fatty Acid Profile

The identified fatty acids by gas chromatography are presented in Table 5. The fatty acids of the Brazil nut (BN) mostly consisted of unsaturated fatty acids (UFA), more than 70% of total fatty acids, mainly linoleic acid (36.9%) and oleic acid (36.4%). The rest of the fatty acids consisted of palmitic acid (15.9%), stearic acid (10.32%), and in minimal amounts, palmitoleic acid (0.24%) and arachidic acid (0.1%). This fatty acid profile of the BN was similar to those reported in the literature [48]. Moreover, it was observed that the fatty acid profile of the Brazil nut (BN), WBM fractions, and cake, did not show a significant difference (*p* < 0.05), demonstrating that the hydrothermal grinding (homogenization) to obtain the beverage from BN does not affect the percentage composition of the fatty acid between raw material and its beverages (WBM and BM-S) and by-products (BM-D, BM-D and cake).

Regarding the beverages, the importance of fatty acids falls mainly on WBM beverage due to the level of total lipids they contain (5.24/100 g f.w.), something that minimally influences the BM-S beverage, which contains only 0.25/100 g f.w. in total lipids. The predominant level of unsaturated fatty acids (MUFA and PUFA) compared to saturated is common in vegetable milk alternatives, as is the case of soy, rice, almond, and cashew milk alternatives, which usually contain more than 70% UFA of the total fatty acids, with the exception of coconut milk, with a predominance of saturated fat [4]. This feature of UFA is considered beneficial for potential health-promoting effects, such as reducing LDL cholesterol, increasing HDL, and controlling cardiovascular events [52]. However, due to the oxidation susceptibility and hydrolytic rancidity of the UFA in the beverage obtained from BN, it is necessary to control the oxidative stabilization processes. Considering the above and the composition of fatty acids (Table 5), the cake and BM-F by-products are food ingredients with “healthy fat” characteristics.

#### 3.4.2. Free Fatty Acids and Monoglycerides

Free fatty acids (FFAs) and mono- and diglycerides are associated with shelf life and quality of edible oils and are normally removed by refining processes because they are susceptible to autooxidation. In the case of vegetable milks the rancidification phenomenon is attributed to lipolysis of triglycerides and increase of FFA, causing off flavor and the deterioration of the beverage [53]. As expected, our work observed corresponding FFA content (Table 6) and total lipid content (Table 1), in the samples.

The FFA concentration of BN (raw material), expressed in oil basis, was around 0.6/100 g oil, consistent with one previously reported, in a range of 0.2–0.8/100 g oil [54], a value within the quality standards established by the European community (≤2/100 g oil, expressed in oleic acid) to be considered as virgin oil [55]. When the FFA of BN and its beverages (WBM and BM-S, with 1 and 1.24/100 g oil, respectively) were compared, there was no significant difference (p ≤ 0.05). However, the slight increase observed can be attributed to triglyceride lipolysis that might occur during the homogenization process, when subjected to high temperatures (75 °C) in an aqueous medium.

Although FFA concentration is considered a promising quality indicator for vegetable milks, reports are scarce. The FFA obtained for the WBM and BM-S beverage had a concentration of 54.9 and 3.11 mg/100 g f.w., respectively, a lower value than almond milk (around 380 mg/100 mL)[29] and soybean milk (99 mg/100 mL)[56], as previously reported. As for the by-products (BM-D, BM-F fraction, and cake), there was also no significant difference with the raw material in terms of FFA concentration (on oil basis), which ranged from 0.18–1.24 mg/g oil, showing that they were not affected by the hydrothermal process of beverage production.

In relation to monoglycerides, expressed in oil bases, the BN had 0.19/100 g oil, comparable to most oilseeds in the range of 0.2–0.4/100 g oil. However, a significant increase (*p* < 0.05) in monoglycerides was observed between the BN (raw material) and beverages (WBM and BM-S, both with 2.2/100 g oil), which suggests the occurrence of lipid hydrolysis during beverage processing. This can be attributed to a reaction with the water molecules, oxygen, exposure to high temperature (75 °C), and, to a lesser extent, by enzymatic hydrolysis (a considerable part would be inactivated by heat treatment).

As for the by-products, a significant difference (*p* < 0.05) was found in the concentration of monoglycerides. Despite the cake coming from the hot water grinding stage (homogenization), its monoglyceride content was low (0.15/100 g oil) with around 2/100 g oil. This can be explained by the protective and barrier effect of the matrix structure of the cake, which contains within it the lipid molecules that were not released in the crushing, thus avoiding contact and reaction with agents that promote lipolysis.

#### 3.4.3. Phytosterols, Tocopherols, and Squalene

These compounds are found in minor quantities, mainly in edible oils of plant origin, appreciated for their antioxidant capacity and biological properties. Therefore, it was considered of interest to evaluate the composition of these minor compounds in the Brazil nut, its derived beverage (WBM and BM-S), and by-products (BM-D, BM-F and cake). According to the results obtained in Table 6, the BN had a total tocopherol content of 18.9 mg/100 g f.w., mainly comprised of γ-tocopherol (16.5 mg/100 g f.w.) and, to a lower extent, by α-tocopherol (2.3 mg/100 g d.w.). It is interesting to remark that the γ-tocopherol content obtained from BN (28.3 mg/100 g oil) may be higher than other edible oils, such as sunflower oil (1.4–4.5 mg/100 g oil) and olive oil (0.9–1.3 mg/100 g oil), which would be an interesting feature considering that γ-Tocopherol is recognized as a potent inhibitor of free radicals against lipid oxidation [57]. Regarding the content of β-sitosterol, the BN had 102.8 mg/100 g oil, which was consistent with a previous report but shows a relatively low level if compared to other nuts (range of 97–468 mg/100 g oil) [58]. On the other hand, the most abundant lipid bioactive minor component found in BN lipid was squalene with 371.68 mg/100 g oil, a great value compared to most other plant sources [59].

The hydrothermal process for the production of the beverage from BN, according to the results of Table 6, in an oil base, had a positive effect on the increase of total tocopherol and β-sitosterol in the beverages (WBM and BM-S) with respect to the raw material. This increase could be linked to the extractability power of the hydrothermal process, hydrolysis of conjugated (esterified) forms, their affinity to emulsion formation, and their hydrophobic character as glycosylated sterols. In the literature, despite being scarce, the WBM beverage (with 3.1 mg/100 g f.w.) is suggested have a total tocopherol comparable to the range reported in vegetable milks, roughly 0.5–3.9 mg/100 mL [5]. Although there are no reports of squalene content in vegetable milks, its high content in BN compared to other vegetable sources (explained in the previous paragraph) could consequently estimate that the WBM beverage (with 14.4 mg/100 g f.w.) contains a higher squalene concentration than other vegetable milks.

In relation to by-products, according to Table 6 and expressed on a dry basis, BM-F has the highest content of lipophilic bioactive minor components, followed by cake and BM-D, which are consistent with the lipid content of each sample (Table 1). In oil bases, no significant differences of these minor components were observed among the by-products, nor within the raw material (BN), which reiterates their stability after beverage processing. In a previous report, using cake (beverage by-product) from the Peruvian Brazil nut, a similar composition was also found: total tocopherol (56.6 mg/100 g oil) and β-sitosterol (183.9 mg/100 g oil) and squalene (531.7 mg/100 g oil) [60]. In general, it is possible to say that the lipophilic bioactive components of the beverage by-products are stable against the hydrothermal process (homogenization stage) and BM-F and cake contain a substantial level.

### 3.5. Characterization of Proteins in Brazil Nut and Derivatives

**Figure 3:**
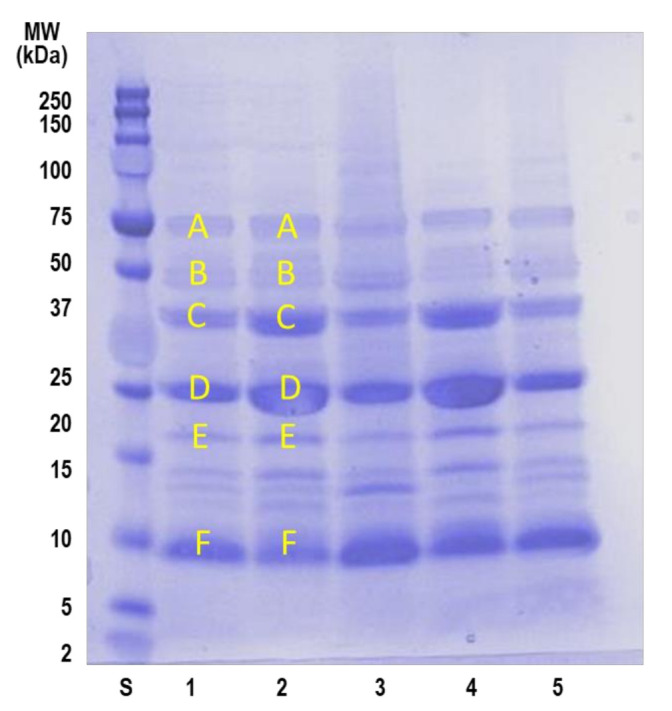
SDS-PAGE profile of Brazil nut products. Lane 1: Brazil nut (*Bertholletia excelsa* HBK); Lane 2: BN protein isolate; Lane 3: BM-D; Lane 4: BM-S; Lane 5: Cake. A to F bands in lanes 1 and 2 were in-gel digested for identification by MALDI TOF/TOF.

The Brazil nut and its derivatives had a significant content of proteins, except for the BM-F. In dry weight (*w*/*w*), BN had 16%, BM-S 40.5%, BM-D 57%, and cake 17% of protein. This remarkable protein level encouraged us to characterize this fraction, which was performed by SDS-PAGE electrophoresis and mass spectrometry of the protein bands using MALDI TOF/TOF analysis. Figure 3 show the electrophoretic profile of the different proteins characterized by the BN and its derivatives.

The main bands appeared in all samples analyzed and corresponded to molecular weights between 75–10 kDa. Mass spectrometry identification after in-gel digestion of the proteins enabled the assignment of three different bands to 11S globulin (B, C, and D band), which points to the occurrence of different electrophoretic forms of this protein (see Table 7). In addition, E and F bands could be putatively assigned to the two reported chains of 2S sulfur-rich storage protein and 2S albumin, although identification must be contrasted by other methods. Similarly, band A, with the highest molecular weight but with the lowest occurrence, matched with convicilin from *Pisum sativum* (Garden pea). The proteins identified and the observed occurrence is comparable to previous reports where 11S protein was found to be the most abundant protein with around 60–70% of the total protein content of the BN, followed by 2S (19–30%) and 7S (8–10%) [61]. Furthermore, in Figure 3, all products kept a characteristic profile, but BM-S (Lane 4) showed a higher band intensity above 20 kDa, suggesting a higher concentration of convicilin and 11S globulin protein than BM-D (Lane 3) and cake (Lane 5). These proteins have been found to be soluble at a neutral and alkaline pH, which explains this behavior [62]. Among Brazil nut proteins, a 9 kDa methionine-rich 2S albumin has been identified as the major allergen in this nut [63]. This protein could correspond with the electrophoretic band F present in all products. Several experiments are in progress to evaluate the resistance of these proteins to gastrointestinal digestion and to identify potential allergenic protein fragments.

### 3.6. Selenium and Other Minerals

#### 3.6.1. Selenium Content

The Brazil nut is recognized as a food with extraordinary concentrations of Selenium (Se), an essential micromineral that performs various physiological functions and is attributed with favorable health effects [3]. In the present study, the Se content of the Brazil nut (BN), its beverages (B-S), and by-products (BM-D, BM-F, and cake) was determined by ICP-MS (Table 8). Brazil nuts had a Se content of 36.28 µg/g f.w. This value was within the range of 10–78 μg/g f.w. of the Se content previously reported [64].

Concerning the BN beverages, the WBM showed a higher Se content (15 µg/100 g f.w.) than BM-S (8.3 µg/100g f.w.) This difference can be attributed mainly to the fact that the WBM has a higher protein content, roughly double that of BM-S, a nutrient associated with selenoamino acids. Another factor that contributed to the Se level of WBM was the participation of BM-D, with 537 µg Se/100 g d.w. According to the Food and Nutrient Database of USDA [65], commercial vegetable milks (soybean, almond, rice, and coconut milk) register Se levels up to a maximum of 2.3 µg/100 g f.w., values much lower than those registered in the WBM and BM-S beverage. However, some previous reports on BN beverages showed higher Se levels than our study. Sartori et al. [9] obtained 106 μg Se /100 mL, using a BN with Se content of 871.4 µg/100 g f.w. and defatting the BN before the beverage is produced. The authors used a higher raw material to water ratio (1:2, *w*/*v*) than in our work (1:7, *w*/*v*). In another study, when BN beverage powder (obtained by spray-drying) was dissolved in water (10 g in 100 mL), the Se concentration was 120 μg/100 mL. This study had also used defatted BN and raw material to water ratio of 1:2 (*w*/*v*) [8]. Considering the above, the raw materials and technological processes employed are factors that influenced the Se level of our beverages compared to others previously reported.

The presence of Selenium linked to proteins in Brazil nuts is well known in the literature. Still, it is interesting to note the difference in Se level between BN (3721 µg/100 g d.w.) and cake (229 µg/100 g d.w.) observed in the present study after beverage processing, even though both have a similar protein content (about 16%, *w*/*w*, dry weight). This variation could be explained by the solubility properties of proteins and its selenoamino acids. According to protein identification (Table 7), albumin proteins (2S albumin, 2S SP1 and 2SP2) are known to be more soluble in aqueous media than 11S globulin and convicilin (soluble in saline solution). In addition, the albumin protein of BN (about 30% of the total protein) is considered to be the most abundant of the selenoamino acids (mainly methionine and cysteine amino acids) [66].

Our work suggests that one BN (~5 g f.w.) could provide about 260% of the dietary reference value (DRV) (70 μg Se/day); and a cup (~240 mL) of BM and BM-S beverages can contribute around 50 and 30% of DRV, respectively. In by-products, the Se level of cake and BM-D in 10 g d.w. are able to provide 32.7 and 76.7% of DVR. To this important contribution of Se from BN and its beverages must be added its recognized good bioavailability, due to its occurrence in organic form. According to Lima [64], by X-ray absorption near edge structure (XANES) spectra study, the Se contained in BN an average of 91% was present in its organic form, detected as C-Se-C compounds. This characteristic of Se and its recognized biological value in health allow BN and the derivatives studied (beverages and by-product with remarkable Se level) to be considered as an important source of this mineral and its possible application in food as a healthy ingredient.

#### 3.6.2. Other Minerals Content

Table 8 show the other minerals quantified in the Brazil nut and its derivatives, omitting from Selenium. In the BN, the content of macrominerals showed the following order of predominance: P (686.05 mg/100 g f.w.), K (576.21 mg/100 g f.w.), Mg (468.85 mg/100 g f.w.), and Ca (186.83 mg/100 g f.w.). In microminerals, those that stood out are: Zn (4.72 mg/100 g f.w.), Fe (3.12 mg/100 g f.w.), and Cu (1.83 mg/100 g f.w.). The values obtained in the present study are similar to those reported by the USDA National Nutrient Database [65] regarding Brazil nuts. Likewise, taking into account the report of dietary reference values (DRV) for nutrients by EFSA [67], for adults over 18 years old, one edible portion of BN (~6 units and 30 g) could provide about 40% of the daily requirement in Mg, P, and Cu, followed by Zn (12.3%), Fe (8.5%), Mn (7.5%), and about 5% in Ca and K.

**Table 8:** Mineral content of Brazil nut (*Bertholletia excelsa* HBK), its beverages (WBM and BM-S), and by-products (BM-D, BM-F, and cake).

Minerals (mg/100 g)	Raw Material		Beverages				By-Products						DRV ^3^ (mg/Day)
	BN ^1^		WBM ^1^		BM-S ^1^		BM-D ^2^		BM-F ^2^		Cake ^2^		
Mg	468.85	±25 ^a^	35.89	±1.1 ^b^	11.06	±0.67 ^b^	2301.5	±62 ^c^	19.27	±1.06 ^b^	513.03	±8.8 ^a^	325
K	576.21	±40 ^a^	77.70	±0.7 ^b^	55.13	±1.25 ^b^	1949.6	±108 ^d^	38.92	±1.89 ^b^	421.15	±44 ^c^	3500
Ca	186.83	±2.6 ^a^	13.96	±2.3 ^a^	2.36	±0.27 ^a^	1196	±227 ^b^	7.79	±1.18 ^a^	221.62	±1.6 ^a^	1000
P	686.05	±13 ^a^	60.59	±2.3 ^b^	11.38	±1.16 ^b^	4605	±151 ^c^	23.17	±4.38 ^b^	725.11	±11.6 ^a^	550
Zn	4.72	±0.1 ^a^	0.43	±0.01 ^b^	0.02	±0.00 ^d^	36.4	±0.27 ^e^	0.59	±0.08 ^b^	5.71	±0.1 ^c^	11.5
Cu	1.83	±0.1 ^a^	0.21	±0.0 ^b^	0.16	±0.00 ^b^	3.86	±0.03 ^d^	0.16	±0.02 ^b^	1.48	±0.0 ^c^	1.5
Fe	3.12	±0.1 ^a^	0.22	±0.0 ^bd^	0.01	±0.00 ^d^	19.04	±0.22 ^e^	0.31	±0.05 ^b^	3.40	±0.01 ^c^	11.0
Mn	0.75	±0.1 ^a^	0.07	±0.0 ^b^	0.01	±0.00 ^b^	6.66	±0.14 ^e^	<1.1 ^b^		1.06	±0.02 ^c^	3
Se (μg/100g)	3628	±51 ^a^	15	±0.2 ^d^	8.3	±0.14 ^d^	537	±6 ^b^	19	±0.3 ^d^	229	±3 ^c^	70 (µg/day)

^1^ Expressed on fresh weight basis; ^2^ Expressed on dry weight basis; ^3^ DRV, Dietary reference value based in Population reference intakes and adequate intakes (PRI-AI) of minerals for adults (estimated for males and females ≥18 years old), EFSA [67]; Data are expressed as means ±SD (*n* = 3). The different superscript letters within a row are significantly different (*p* < 0.05).

With respect to beverages, as shown in Table 8, in most minerals, WBM had slightly higher concentrations than BM-S. This is understandable due to the participation of the sediment in the WBM, which has a high level of minerals. When compared with other commercial vegetable milks (from soybean, almond, rice, and coconut), reported by Vanga and Raghavan [68], the WBM showed, in most minerals, a level within the range, i.e., at Mg (13–49 mg/100 g f.w.), K (47–364 mg/100 g f.w.), P (48–108 mg/100 g f.w.), Fe (0.05–0.84 mg/100 g f.w), and Z (0.38–0.75 mg/100 g f.w.). In BM-S, a lower mineral content than commercial vegetable milks was noted. Regarding calcium, both WBM (13.9 mg/100 g f.w.) and BM-S (2.4 mg/100 g f.w.) had lower concentrations than other commercial plant milks, which typically have values greater to 100 mg/100 mL as they are often fortified to mimic the calcium content of cow’s milk [68]. In the aspect of daily nutritional intake (DRV of Table 8), a cup (~240 mL) of WBM beverage may contribute mainly Mg with 26.5%, P with 26.4%, and Cu with 34%. As for the BM-S beverage, only Cu with 26%. The rest of the minerals had a contribution of less than 10% in both WBM and BM-S beverages.

Regarding by-products, it was observed (Table 8) that the BM-D had higher mineral concentrations (*p* < 0.05) than cake and BM-F. Regarding its daily nutritional contribution (according to DRV), an intake of 10 g d.w. of BM-D may to provide P with 84%, Mg with 71%, Zn with 32%, Cu with 27%, Mn with 22%, Fe with 17%, and Ca with 12%. The cake, with an intake of 100 g d.w., may provide Mg with 160%, P with 132%, Cu with 102%, Zn with 50%, Mn with 36%, Fe with 30%, and Ca with 22%. According to the above, BM-D and cake by-products represent an important source of Mg, P, Zn, and Cu and Mn, Fe, and Ca to a lesser degree.

### 3.7. Antioxidant Capacity

In this work, three in vitro methods were used to evaluate the antioxidant capacity of the BN samples, as shown in Table 9. The antioxidant level of the Brazil nut showed consistency to the previous report, i.e., 2.6 μmol TE/g f.w. by DPPH assay [20], ~11 μmol TE/g f.w. by TEAC, and ~26 µmol TE/g f.w. by ORAC [12].

Regarding the BN beverages, WBM and BM-S showed a similar antioxidant capacity, with no significant difference (*p* < 0.05). In the DPPH assay, WBM and BM-S had 8 and 7 μmol TE/100 g f.w., respectively, values that are in the range of other commercial vegetable milks that range from 4.7–30.5 μmol TE/100 mL by DPPH [28].

With respect to the antioxidant capacity of the BN by-products (Table 9), the BM-D showed greater antioxidant power than the cake. These results had a relationship with the TPC content shown in Table 2. In the literature, there are no reports about the antioxidant capacity of the cake obtained as a by-product of beverage processing of BNs, but our result is similar to the cake obtained from defatted BNs by pressing (4.07 μmol TE/g d.w. by TEAC assay), reported by Gomes [30]. If compared to the antioxidant power (by TEAC assay) of the cake produced by soymilk (called “okara”), with 19–29 μmol TE/g d.w. [69], the antioxidant capacity of the BN cake was minimal.

## 4. Conclusions

The present study reports a comprehensive compositional characterization of the Brazil nut (BN), its derived beverages, and processing by-products. The BN showed an interesting nutritional quality related to its bioactive constituents (such as unsaturated fatty acids, phytosterols, tocopherols, squalene, phenolic compounds, proteins, and selenium). Twenty-four phenolic compounds were identified in Brazil nuts and products, flavan-3-ols and the hydroxybenzoic acids, the phenolics more abundant. Brazil nut beverages showed an interesting content in phenolic compounds, and their nutritional value was comparable to other commercialized vegetable milks, WBM being the beverage with the higher content of nutritional components as the minor bioactive lipids (total tocopherols, β-sitosterol, and squalene). The selenium content of the Brazil nut beverage was higher than those reported for other commercial vegetable milks. For this reason, the BN beverage represented an important alternative source to the daily requirement of Se in the human diet. Additionally, the hydrothermal processing for beverage production has a significant effect on the content of some minor lipid components (FFA, MG, total tocopherol, β-sitosterol, and squalene), by the hydrolysis of triglycerides and certain conjugated compounds. Regarding the BN by-products, the fat fraction (BM-F) is mainly composed of oleic and linoleic acids and other minor lipid components; the sediment fraction, a by-product produced in minimal quantities, showed high content of protein, selenium, other minerals, and phenolic compounds; and the cake retained many of the nutritional components of the BNs. The present study provides some support for future studies related to the improvement of the technological processes used to produce BN beverages, to influence the stability of these processes (physical and oxidative) regarding bioactive compounds present in the beverage, as well as the development of a smart formulation of BN beverages according to the target consumer (older people, children, others).

## Figures and Tables

**Table 1:** Centesimal composition and physicochemical properties of the Brazil nut (*Bertholletia excelsa* HBK), its beverages, and by-products.

Characteristic	Raw Material	Beverages		By-Products		
	BN ^1^	WBM *^,1^	BM-S ^1^	BM-D ^2^	BM-F ^2^	Cake ^2^
Protein	16.03	1.43	0.80	56.88	0.65	16.9
Lipid	58.52	5.24	0.25	14.6	81.3	44.2
Carbohydrate	19.61	1.94	0.83	16.65	16.29	35.45
Ashes	3.35	0.33	0.10	11.87	1.76	3.5
Energy (Kcal)	669.2	60.6	8.8	425.5	799.5	607.2
Total solids ^1^	97.51	8.93	1.98	31.41	49.21	36.17
Acidity ^3^	0.18	0.03	0.03	0.54	-	0.1
pH	6.4	6.5	6.5	6.7	-	6.6
Soluble solid (°Brix)	14.5	2.6	3.0	4.8	-	6.8

Abb.: Brazil nut (BN), Whole beverage (WBM), Water soluble fraction (BM-S), Sediment fraction (BM-D). * Values of WBM were estimated from the fractions; ^1^ Expressed as g/100 fresh weight (f.w.); ^2^ Expressed as g/100 g fry weight (d.w.); ^3^ Expressed as g citric acid/100 g.

**Table 2:** Total phenolic content of the Brazil nut (*Bertholletia excelsa* HBK), its beverages (WBM and BM-S), and by-products (BM-D and cake), expressed in mg GAE/100 g.

Samples		TPC			
		Dry Weight		Fresh Weight	
Raw material	BN	108.0	±3.9 ^b^	105.3	±3.8 ^a^
Beverages	WBM *	79.5	±1.5 ^b^	7.1	±0.1 ^c^
	BM-S	314.0	±5.2 ^a^	6.2	±0.1 ^c^
By-products	BM-D	83.4	±3.1 ^b^	28.7	±1.1 ^b^
	Cake	49.2	±2.7 ^c^	17.8	±1.0 ^b^

Data are expressed as means ± SD (*n* = 3). Values with different superscript letters in the column are significantly different (*p* < 0.05); * Value of WBM was estimated from their fractions.

**Table 4:** Individual phenolic content (µg/g) in Brazil nut (*Bertholletia excelsa* HBK), its beverages (WBM and BM-S) and by-products (BM-D and cake) by HPLC-DAD-MS.

Rt (Min)	Phenolic Compound	BN ^1^		WBM *^,1^		BM-S ^1^		BM-D ^2^		Cake ^2^	
	Phenolic Acids										
6.4	Gallic acid	6.3	±0.2	0.7	±0.0	0.5	±0.0	17.7	±0.8	6.4	±0.3
6.9	Gallic acid derivative	6.9	±0.3	0.6	±0.0	0.4	±0.0	17.7	±0.2	6.5	±0.3
21.7	Protocatechuic acid derivative	42.0	±1.9	3.0	±0.1	2.1	±0.1	83.2	±4.0	31.7	±1.4
28.4	4-hydroxybenzoic acid	6.5	±0.3	0.5	±0.0	0.4	±0.0	3.7	±0.0	2.2	±0.1
30.2	Vanillic acid	18.0	±0.8	1.5	±0.0	1.4	±0.0	4.9	±0.1	3.3	±0.1
35.4	p-Coumaric acid	8.5	±0.3	0.9	±0.0	0.8	±0.0	4.9	±0.1	2.6	±0.1
36.4	Ferulic acid	4.6	±0.2	0.5	±0.0	0.5	±0.0	1.7	±0.1	1.0	±0.0
42.1	Vanillic acid derivative	1.6	±0.1	0.2	±0.0	0.2	±0.0	0.6	±0.0	0.5	±0.0
37.9	Ellagic acid derivative	45.4	±0.4	4.7	±0.1	3.3	±0.1	138.6	±3.8	49.1	±1.7
39.7	Ellagic acid	36.2	±1.4	3.5	±0.0	2.1	±0.0	134.1	±3.4	46.8	±2.5
	*Phenolic aldehyde*										
32.4	Vanillin	5.6	±0.2	0.5	±0.0	0.5	±0.0	1.3	±0.1	1.2	±0.0
	*Flavonoids*										
22.3	Catechin	175.4	±7.9	21.6	±0.5	20.2	±0.5	127.6	±3.0	79.7	±3.3
24.0	Catechin derivative	86.1	±2.6	9.2	±0.5	8.9	±0.5	25.7	±1.2	18.3	±0.9
30.9	Epicatechin	21.8	±0.7	1.5	±0.0	1.4	±0.0	16.2	±0.4	8.0	±0.1
33.4	Catechin-gallate	71.0	±1.7	8.5	±0.1	8.2	±0.1	24.8	±0.6	16.7	±0.3
33.8	Epicatechin-gallate	14.3	±0.4	1.4	±0.0	1.3	±0.0	13.6	±0.2	6.3	±0.2
34.6	Epigallocatechin 3-O-gallate	7.4	±0.3	0.3	±0.0	0.2	±0.0	11.0	±0.3	4.3	±0.1
37.0	Taxolin (dihydroquercetin)	--		--		--		--		--	
38.9	Quercetin	6.7	±0.2	0.9	±0.0	0.9	±0.0	0.2	±0.0	1.0	±0.0
40.0	Myricetin-3-O-rhamnoside	10.6	±0.5	0.2	±0.0	0.2	±0.0	6.8	±0.4	3.6	±0.0
	*Total phenolic acids*	176.1	±5.8	16.0	±0.4	11.8	±0.3	407.1	±12.5	150.3	±6.5
	*Phenolic aldehyde*	5.6	±0.2	0.5	±0.0	0.5	±0.0	1.3	±0.1	1.2	±0.0
	*Total flavonoids*	393.4	±14.4	43.7	±1.2	41.1	±1.1	225.9	±6.1	138.0	±5.0
	*Total phenolic compounds*	575.0	±20.4	60.2	±1.6	53.3	±1.4	634.3	±18.6	289.4	±11.5

* Value of WBM was estimated from their fractions; ^1^ Expressed on fresh weight basis.; ^2^ Expressed on dry weight basis.; -- Detected but not quantified.

**Table 5:** Fatty acid content of the Brazin nut (*Bertholletia excelsa* HBK), WBM fractions, and cake.

Fatty Acids (FA) %	BN	WBM Fractions			Cake
		BM-S	BM-D	BM-F	
Palmitic (C16:0)	15.95 ± 1.06 ^a^	15.35 ± 0.15 ^a^	15.36 ± 0.05 ^a^	15.30 ± 0.05 ^a^	15.17 ± 0.11 ^a^
Palmitoleic (C16:1)	0.24 ± 0.01 ^b^	0.20 ± 0.02 ^b^	0.21 ± 0.02 ^b^	0.22 ± 0.02 ^b^	0.22 ± 0.01 ^b^
Stearic (C18:0)	10.32 ± 2.59 ^c^	9.84 ± 0.24 ^c^	10.09 ± 0.15 ^c^	9.57 ± 0.03 ^c^	9.52 ± 0.17 ^c^
Oleic (C18:1)	36.40 ± 5.68 ^d^	36.66 ± 1.23 ^d^	36.59 ± 0.34 ^d^	35.85 ± 0.18 ^d^	35.33 ± 0.45 ^d^
Linoleic (C18:2)	36.99 ± 7.22 ^e^	37.86 ± 1.30 ^e^	37.64 ± 0.47 ^e^	38.97 ± 0.10 ^e^	39.64 ± 0.52 ^e^
Arachidic (C20:0)	0.10 ± 0.06 ^f^	0.09 ± 0.01 ^f^	0.11 ± 0.03 ^f^	0.09 ± 0.01 ^f^	0.11 ± 0.02 ^f^
Total Saturated (SFA)	26.37 ± 3.71 ^g^	25.28 ± 0.39 ^g^	25.56 ± 0.23 ^g^	24.97 ± 0.09 ^g^	24.81 ± 0.30 ^g^
Total Unsaturated (UFA)	73.63 ±12.90 ^h^	74.72 ± 2.55 ^h^	74.44 ± 0.82 ^h^	75.03 ± 0.30 ^h^	75.19 ± 0.98 ^h^
Polyunsaturated (PUFA)	36.99 ± 7.22 ^i^	37.86 ± 1.30 ^i^	37.64 ± 0.47 ^i^	38.97 ± 0.10 ^i^	39.64 ± 0.52 ^i^
Monounsaturated (MUFA)	36.64 ± 5.69 ^j^	36.86 ± 1.25 ^j^	36.80 ± 0.36 ^j^	36.07 ± 0.19 ^j^	35.55 ± 0.46 ^j^

Data are expressed as means ± SD (*n* = 3). Values with different superscript letters in each row are significantly different (*p* < 0.05).

**Table 6:** Free fatty acids, monoglycerides, phytosterols, β-Sitosterol, and squalene content of the Brazil nut (*Bertholletia excelsa* HBK), its beverages (WBM and BM-S), and by-products (BM-D, BM-F, and cake).

Compound		Raw Material		Beverages				Byproducts					
		BN ^1^		WBM ^1^		BM-S ^1^		BM-D ^2^		BM-F ^2^		Cake ^2^	
FFA	(mg/100 g)	346	±61 ^a^	51.3	±12 ^ab^	3.11	±0.7 ^b^	181.4	±41 ^ab^	794	±172 ^c^	80.5	±4.1 ^ab^
	(g/100 g oil)	0.59	±0.10 ^abc^	1.0	±0.2 ^abc^	1.24	±0.3 ^bc^	1.24	±0.28 ^b^	0.98	±0.2 ^abc^	0.18	±0.01 ^a^
Monoglycerides	(mg/100 g)	110.8	±32 ^a^	113.5	±34 ^a^	5.66	±0.8 ^a^	304.3	±65 ^a^	1789	±58 ^b^	66.3	±33 ^a^
	(g/100 g oil)	0.19	±0.05 ^a^	2.20	±0.6 ^b^	2.25	±0.3 ^b^	2.08	±0.45 ^b^	2.20	±0.6 ^b^	0.15	±0.07 ^a^
Total tocopherol	(mg/100 g)	18.9	±6 ^a^	3.1	±0.3 ^c^	0.22	±0.04 ^bc^	7.2	±1.3 ^abc^	47.8	±5 ^d^	25.5	±0.8 ^ae^
	(mg/100 g oil)	32.3	±11 ^a^	59.9	±6.1 ^b^	88.1	±17 ^b^	49.3	±9 ^ab^	58.77	±6 ^ab^	57.6	±1.8 ^ab^
γ-Tocopherol	(mg/100 g)	16.5	±4.6 ^a^	2.4	±0.08 ^b^	0.18	±0.04 ^b^	5.76	±1.1 ^b^	37.4	±1 ^c^	19.9	±1.9 ^a^
	(mg/100 g oil)	28.3	±7 ^ac^	47.1	±1.4 ^abc^	73.5	±14 ^b^	39.5	±7 ^c^	45.98	±1 ^abc^	45.1	±4.3 ^abc^
α-Tocopherol	(mg/100 g)	2.4	±2 ^a^	0.7	±0.26 ^a^	0.04	±0.01 ^a^	1.4	±0.2 ^a^	10.4	±4 ^b^	5.5	±1.1 ^ab^
	(mg/100 g oil)	4.02	±1.3 ^a^	12.8	±4.8 ^a^	14.61	±2.9 ^a^	9.78	±1.5 ^a^	12.79	±2.5 ^a^	12.5	±2.5 ^a^
β-Sitosterol	(mg/100 g)	60.2	±15 ^a^	7.1	±0.9 ^b^	0.73	±0.23 ^b^	30.7	±6 ^ab^	105	±11 ^c^	62.1	±3 ^a^
	(mg/100 g oil)	102.8	±26 ^b^	138.4	±17 ^ab^	289.5	±89 ^a^	210.1	±43 ^ab^	129	±13 ^ab^	140.4	±6 ^ab^
Squalene	(mg/100 g)	217.5	±13 ^a^	13.2	±2.8 ^b^	0.69	±0.11 ^b^	40.1	±11 ^b^	207.6	±41 ^a^	123.6	±8 ^c^
	(mg/100 g oil)	371.7	±23 ^a^	256.7	±50 ^a^	272.62	±43 ^a^	274.8	±74 ^a^	255.4	±50 ^a^	279.7	±18 ^a^

^1^ mg/100 g is expressed on fresh weight basis; ^2^ mg/100 g is expressed on dry weight basis. Data are expressed as means ±SD (*n* = 2). The different superscript letters within a row are significantly different (*p* < 0.05).

**Table 7:** Identification of peptide fragments of Brazil nut (*Bertholletia excelsa* HBK) by in-gel digestion of electrophoretic bands.

Band	Tryptic Peptide (Ion)	Protein Fragment	Sequence	Protein Name	Entry Number
B	1145.44	194–202	RSQKQRGER	11S globulin	Q84ND2 *(Bertholletia excelsa)*
B	1202.43	338–327	MMAPLWRLNA	11S globulin	Q84ND2 *(Bertholletia excelsa)*
B	1145.49	434–442	RLSQEEARR	11S globulin	Q84ND2 *(Bertholletia excelsa)*
B	2256.85	42–60	QYRLEAEAGVSE VWDYTDQ	11S globulin	Q84ND2 *(Bertholletia excelsa)*
C	1202.46	184–193	RHFFLAGNIQ	11S globulin	Q84ND2 *(Bertholletia excelsa)*
C	999.37	90–97	LYYVTQGR	11S globulin	Q84ND2 *(Bertholletia excelsa)*
C	882.39	131–137	QDQHQKV	11S globulin	Q84ND2 *(Bertholletia excelsa)*
C	1202.55	185–194	HFFLAGNIQR	11S globulin	Q84ND2 *(Bertholletia excelsa)*
D	1165.4	367–376	GETVFDDNLR	11S globulin	Q84ND2 *(Bertholletia excelsa)*
D	1229.54	422–433	RGIPVGVLANAY	11S globulin	Q84ND2 *(Bertholletia excelsa)*
D	2315.75	49–70	GVSEVWDYTD QQFRCAGVAAL	11S globulin	Q84ND2 *(Bertholletia excelsa)*

**Table 9:** Antioxidant capacity of the Brazil nut (*Bertholletia excelsa* HBK), its beverages (WBM and BM-S), and by-products (BM-D and cake), expressed as μmol TE/g.

Assay	Raw Material		Beverages				Byproducts			
	BN ^1^		WBM ^1^		BM-S ^1^		BM-D ^2^		Cake ^2^	
DPPH	1.72	±0.11 ^a^	0.08	±0.00 ^b^	0.07	±0.00 ^b^	1.52	±0.04 ^c^	0.27	±0.03 ^d^
TEAC	14.39	±0.46 ^a^	0.82	±0.04 ^b^	0.71	±0.03 ^b^	11.03	±0.89 ^c^	3.34	±0.15 ^d^
ORAC	10.98	±0.45 ^a^	0.61	±0.01 ^b^	0.47	±0.01 ^b^	14.18	±0.77 ^c^	6.73	±0.40 ^d^

^1^ Expressed on fresh weight basis; ^2^ Expressed on dry weight basis. Data are expressed as means ± SD (*n* = 3). The different superscript letters within a row are significantly different (*p* < 0.05).

## Data Availability

Data is contained within the article.

## Supplementary Materials

The following are available online at https://www.mdpi.com/article/10.3390/foods10123007/s1, Figure S1: Calibration curves of the standard phenolic compounds (catechin, epicatechin, quercetin and rutin), Figure S2: Calibration curves of the standard phenolic compounds (gallic acid, 4-hidroxy benzoic acid, vanillic acid, p-coumaric acid, ferulic acid and ellagic acid) for HPLC quantitation.
