# Supplementary material for: Composition of Brazil Nut (Bertholletia excels HBK), Its Beverage and By-Products: A Healthy Food and Potential Source of Ingredients
Source: Foods. 2021 Dec 4;10(12):3007. doi: 10.3390/foods10123007 (PMC8700994; doi:10.3390/foods10123007)
Supplement: Supplementary file 1 [file foods-10-03007-s001.zip › foods-1476916-SI.pdf]

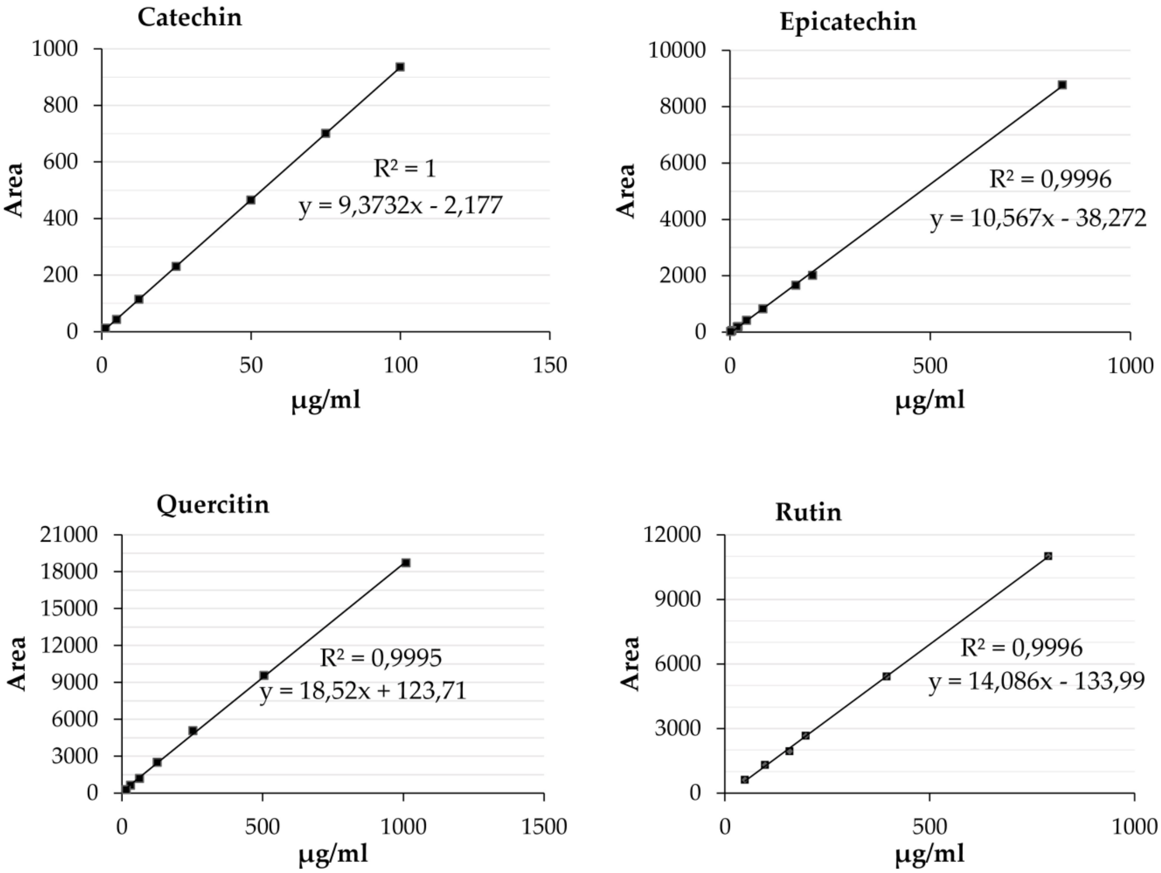

Figure S1. Calibration curves of the standard phenolic compounds (catechin, epicatechin, quercetin and rutin) for HPLC quantitation.

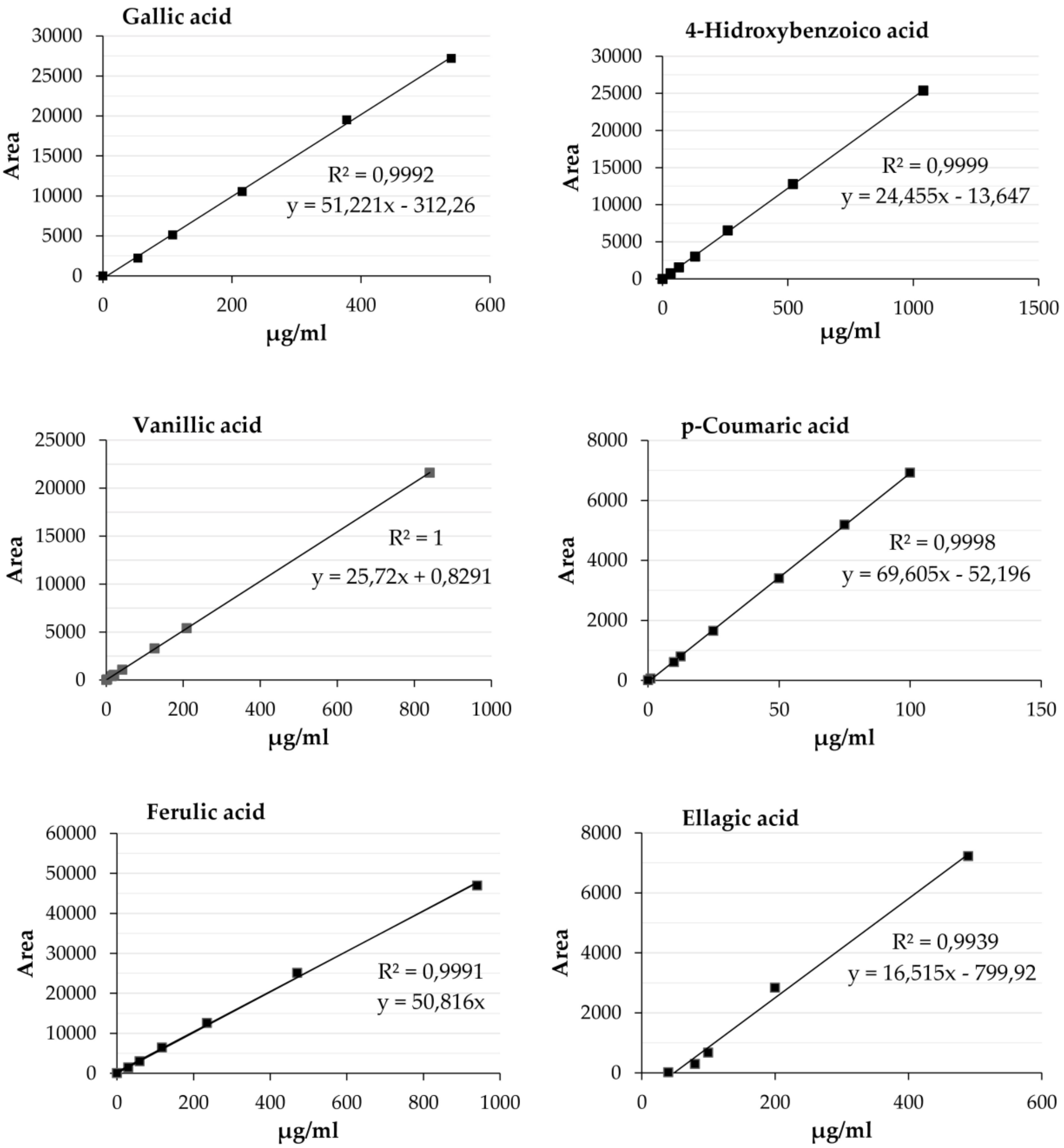

Figure S2. Calibration curves of the standard phenolic compounds (gallic acid, 4-hidroxy benzoic acid, vanillic acid, p-coumaric acid, ferulic acid and ellagic acid) for HPLC quantitation.
